# Determinants of Poor Adherence to Anti-Tuberculosis Treatment in Mumbai, India

**Published:** 2010

**Authors:** Suparna Bagchi, Guirish Ambe, Nalini Sathiakumar

**Affiliations:** 1 Georgia Division of Public Health, USA; 2 Executive Health Officer, Brighan Mumbai Municipal Corporation. Mumbai, India; 3 University of Alabama at Birmingham, Birmingham, USA

**Keywords:** Tuberculosis, DOTS, Mumbai, Adherence, smoking, drug supply

## Abstract

**Objectives:**

In this study, we investigated the determinants of poor adherence with anti-tuberculosis therapy among pulmonary tuberculosis (TB) patients in Mumbai, India, receiving Directly Observed Treatment Short Course (DOTS) therapy.

**Methods:**

A cross-sectional study on 538 patients receiving DOTS I and II regimen was conducted. Patients were interviewed and clinical and laboratory data were collected. Eighty seven patients were considered non-adherent. Multivariable logistic regression was used to determine risk factors associated with non-adherence.

**Results:**

Factors associated with non-adherence were found to be different among the newly-diagnosed patients and all the other residual groups. Smoking during treatment and travel-related cost factors were significantly associated with non-adherence in the newly-diagnosed patients, while alcohol consumption and short-age of drugs were significant in the residual groups.

**Conclusions:**

An approach, targeting easier access to drugs, an ensured drug supply, effective solutions for travel-related concerns and modification of smoking and alcohol related behaviors are essential for treatment adherence.

## INTRODUCTION

The World Health Organization (WHO) has estimated that about 8 billion people worldwide are infected with *Mycobacterium tuberculosis* and each year 1.87 million people die of Tuberculosis (TB).^1^ India bears approximately 30% of the world’s burden of TB, with an estimated incidence of 85 per 100,000 new smear positive cases.^2^

In the 1960s, India initiated a National Tuberculosis Programme (NTP) to combat TB. Expert reviews undertaken by Indian government indicated that less than 30% of patients enrolled completed the treatment.^3^ Major reasons identified for poor completion rates included shortage of drugs, inadequate staff and poor patient follow-up.^3^ In 1993, the NTP incorporated WHO recommended Directly Observed Treatment Short Course (DOTS) global strategy, and was known as the “Revised National Tuberculosis Control Programme (RNTCP)”.^4^–^6^ India is currently the second largest DOTS provider in the world.^4^–^6^

Poor patient adherence to the treatment regimen is a major cause of treatment failure and of the emergence of drug-resistant TB. Previous research reported travel expenses, traveling to treatment centers, male sex, poor patient information and communication, alcoholism and homelessness as the major determinants of non-adherence to anti-TB treatment.^7^–^15^ Patient adherence to the standard anti-TB therapy in developing countries has been estimated to be as low as 40%.^16^ The present study was undertaken at the DOTS centers in Mumbai, India, to determine the extent of adherence in pulmonary TB patients receiving DOTS therapy and to evaluate the factors contributing to non-adherence.

## METHODS

Prior to the study, HSRB approval was taken from the IRB committee of the University of Alabama, Birmingham and the ethics committee of Brihan Mumbai Municipal Corporation, Mumbai.

### Study site

**Figure 1:**
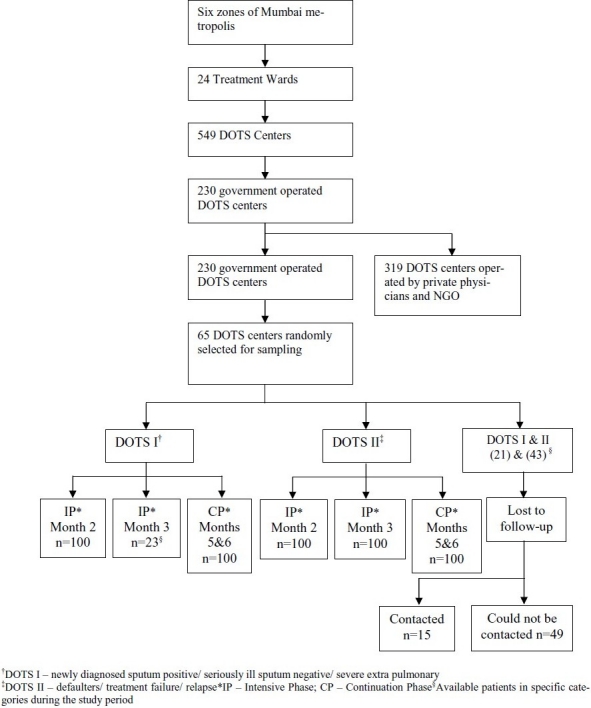
Subject recruitment among the intensive phase (IP) and the continuous phase (CP) of DOTS I and II regimens

The study sites were government operated DOTS centers in Mumbai, India during February to April 2003 (Figure 1).

#### Patient enrollment at DOTS centers

When a patient is diagnosed with TB, he/she is referred to the DOTS center closest to his/her residence. At the DOTS center, the patient is registered; a treatment card and a patient identity card are developed. The treatment card con-tains information on patient’s demographic, treatment history, dates associated with current DOTS treatment and are maintained at the DOTS center. The patient identity card contains information on patient’s current treatment and is carried by the patient. It is updated by the DOTS center staff. The physician at the DOTS center speaks with the patient and his/her family, emphasizing the importance of adhering to the treatment schedule.

Under the DOTS program of RNTCP, after a positive diagnosis of pulmonary TB, the patient is categorized to receive a particular drug regimen (Category I, II, or III) based on the results of laboratory diagnosis and past history of TB. All the three categories of treatment consist of two phases of treatment: intensive and continuous phases. During the intensive phase, the pa-tient comes to the DOTS center three times a week and receives drugs under direct supervision. During the continuous phase, the patient comes to the DOTS center once a week; receives one dose under supervision and carries home the remaining doses for the week. The above process is continued until the end of the treatment regimen. If the patient continues to be sputum positive at the end of the intensive phase, an additional month of intensive treatment is provided.

### Design

The study was cross-sectional and was designed to determine factors that contribute to non-adherence to the anti-TB treatment. In this study, we defined non-adherence as any patient belonging to either intensive or continuation phase of either category I or II who missed one week of treatment in a month (either consecutive or sporadic doses totaling a week). The study was conducted from February through April, 2003. Subject inclusion and exclusion criteria are provided in Table 1.

**Table 1:** Patient selection criteria for the study

**Inclusion criteria**	•	Men and women > 19 years of age receiving anti-TB treatment at the DOTS centers
	•	Confirmed pulmonary TB patients by laboratory investigations (sputum for acid-fast bacilli status, chest X-ray)
	•	Receiving either Category I or II drug regimen
**Exclusion criteria**	•	Patients < 20 years of age
	•	Sputum negative patients receiving Category I drug regimen
	•	Pulmonary TB patients receiving Category III DOTS regimen
	•	Pregnant and extra pulmonary TB patients
	•	Patients too ill to be interviewed

### Subject selection

Power calculations, based on non-adherence reported in previous studies conducted in India (6% and 11%) indicated that a sample size of 100 patients within each subgroup would have 80% power to detect a statistically significant 19% difference in risk factors between the adherent and non-adherent groups.^17^ With the exception of two groups (DOTS I with additional third month of intensive therapy and DOTS I and II lost to follow-up group), we recruited 100 patients in each group. We were unable to recruit 100 patients in the above two groups because of the small numbers of patients in these groups and difficulty of tracing these patients as they had either moved their residence or were not available in their homes. Of the total number of 587 subjects initially selected, 49 subjects belonging to the DOTS I and II lost to follow-up group could not be traced. Of the remaining 538 patients, 536 participated (partici-pation rate of 99.6%). Two subjects receiving intensive therapy of the DOTS II regimen refused to participate and were replaced.

### Interviews

Eleven research assistants trained by one of the investigators (SB) conducted the interviews. Research staff explained the purpose of the study, confidentiality of data and recruited those who agreed to participate in the study. Informed consent was obtained before data collection. Subjects received a gift package, irrespective of their decision to participate. Interviews were conducted in the national (Hindi) or the local (Marathi) language. The questionnaire, developed to ascertain potential risk factors of non-adherence consisted of a combination of open and close-ended questions that elicited information on patient-related (socio-demographic, knowledge and attitude about TB), travel-related (concerns related to traveling to health center to collect the medicines) and patient/health care provider related factors (communication with health care staff). We obtained data on non-adherence to treatment schedule from patient treatment cards to specify the adherence/non-adherence status. Data on potential risk factors were based on self-reports.

### Analysis

Subjects were classified either as adherent or non-adherent based on the study criteria. We compared the prevalence of each risk factor between the adherent and non-adherent group using the prevalence odds ratios (POR) with its corresponding 95% confidence interval. Logistic regression procedures were used to adjust for multiple risk factors. We included a risk factor in the logistic regression model if there was a moderate association (POR ≥ 2.0 or ≤ 0.5) between the risk factor and non-adherence or if a risk factor was statistically significant associated with non-adherence. Factors significantly associated with non-adherence were separately entered into multivariable logistic regression models for the newly-diagnosed and other group.

## RESULTS

Of the 538 patients, 451(84%) were adherent and 87 (16%) were non-adherent. Men comprised two-thirds (64%) of the study group, and women one-third (36%). Men and women had non-adherence rates of 18% (63/34) and 12% (24/194), respectively. The median age of the study group was 30 years (Table 2). About 80% of subjects belonged to the Hindu religion. Most subjects (92%) lived at their current residence for more than two years, and about 78% had less than three members in the household. Two-thirds of the subjects were literate, and about 40% were employed. Differences in socio-demographic characteristics between the adherent and non-adherent groups were not remarkable. We examined the distribution of risk facors and crude PORs for non-adherence foreach of the seven subgroups of patients, and found that Category I patients receiving second month of intensive phase treatment were distinctly different compared to the other six subgroups. Therefore, further analyses are presented for two groups: newly-diagnosed group and the residual subjects (henceforth referred to as the “residual other” group).

**Table 2:** Socio-demographic characteristics of the study group according to adherence/non-adherence status

CHARACTERISTIC	Adherent		Non-adherent			Total	
	N	%	N	%	p*	N	%
**Total**	451	100	87	100		538	100
**Age (years)**							
≤ 30	247	55	39	53		286	45
> 30	204	45	48	47	0.09	252	55
Median	31		30			30	
**Gender**							
Male	281	62	63	72		344	64
Female	170	38	24	28	0.07	194	36
**Marital status**							
Married	291	65	57	65		348	66
Other	160	35	30	35	0.86	190	34
**Religion**							
Hindu	350	78	70	78		420	80
Other	101	22	17	22	0.56	118	20
**Duration at present residence**							
≤ 2 yrs	52	12	7	11		59	8
> 2 yrs	399	88	80	89	0.34	479	92
**Literacy**							
Illiterate‡	145	32	35	40		180	33
Literate	305	68	52	60	0.14	357	67
**Employment status**							
Unemployed	255	57	51	57		306	59
Employed	196	43	36	43	0.71	232	41
**Household members**							
0-3	347	77	74	85		421	78
>3	104	23	13	15	0.09	117	22

* Chi-square values

‡ Less than 6 years of schooling

Table 3 presents the socio-demographic char-acteristics of the newly-diagnosed and the residual other group according to the adherence/non-adherence status. Among the newly-diagnosed group, there were no remarkable differences in socio-demographic characteristics between the adherent and non-adherent groups. Among the residual other group, men were more likely to be non-adherent compared to women.

**Table 3:** Socio-demographic characteristics of the newly-diagnosed and the other residual group according to the adherent/non-adherent status

Characteristic	Newly-diagnosed					Other				
	
	Adherent		Non-adherent			Adherent		Non-adherent		
	
	N	(%)	N	(%)	p*	N	(%)	N	(%)	p*
**Total**	87	(87)	13	(13)		364	(83)	74	(17)	
**Age (years)**										
< 30	30	(34)	6	(46)		190	(52)	32	(43)	
≥ 30	57	(66)	7	(54)	0.44	174	(48)	42	(57)	0.16
**Gender**										
Female	34	(39)	6	(46)		136	(37)	18	(24)	
Male	53	(61)	7	(54)	0.63	228	(63)	56	(76)	0.03
**Marital status**										
Other	36	(41)	2	(15)		124	(34)	28	(38)	
Married	51	(58)	11	(85)	0.12	240	(66)	46	(62)	0.53
**Religion**										
Others	20	(23)	3	(23)		81	(22)	14	(19)	
Hindu	67	(77)	10	(77)	1.0	283	(78)	60	(81)	0.53
**Duration at present residence**										
> 2 yrs	13	(15)	0	(0)		325	(89)	67	(91)	
≤ 2yrs	74	(85)	13	(100)	0.11	39	(10)	7	(9)	0.75
**Literacy**										
Literate	63	(72)	10	(77)		242	(67)	42	(57)	
Illiterate	24	(28)	3	(23)	0.74	121	(33)	32	(43)	0.11
**Employment status**										
Employed	38	(44)	4	(31)		206	(57)	42	(57)	
Unemployed	49	(56)	9	(69)	0.42	158	(43)	62	(43)	0.98
**Household members**										
0-3	70	(80)	12	(92)		277	(76)	62	(84)	
>3	17	(20)	1	(8)	0.31	87	(24)	12	(16)	0.15

* Chi-square values.

Table 4 provides crude PORs for non-adherence by potential risk factors among the newly-diagnosed and the residual other groups. Among the newly-diagnosed group, six risk factors were found to be significantly associated with non-adherence to treatment. These factors were as follows: smoking during treatment and various types of travel-related factors. Among the residual other group, smoking during treatment and alcohol consumption during the treatment were the main lifestyle-related factors associated with non-adherence. Missing treat-ment due to lack of drug availability was also significantly associated with non-adherence to the treatment in this group.

**Table 4:** Crude prevalence odds ratios (PORs) and 95% confidence intervals for non-adherence by potential risk factors, by group

Characteristics	Newly-diagnosed			Other		
	
	N/T*	POR	95% C.I.	N/T*	POR	95% C.I.
**Demographic factors**						
Gender						
Female	6/40	1.0		18/154	1.0	
Male	7/60	0.7	0.2-2.4	56/284	1.9	1.0-3.6
Household members						
0-3	12/82	1.0		62/339	1.0	
>3	1/18	0.3	0.0-2.7	12/99	0.7	0.3-1.3
**Lifestyle factors**						
Smoking status						
Never	10/94	1.0		62/398	1.0	
Ever	3/6	8.2	1.4-46	12/40	2.4	1.2-5.1
Tobacco chewing						
Never	8/75	1.0		53/329	1.0	
Ever	5/25	2.0	0.6-6.9	21/109	1.3	0.7-2.3
Alcohol use						
Never	12/94	1.0		59/403	1.0	
Ever	1/6	1.3	0.1-12.4	15/35	4.8	2.2-10.3
**Attitude and belief**						
Hide disease from family						
No	11/80	1.0		69/381	1.0	
Yes	2/20	0.6	0.1-3.1	5/57	0.5	0.2-1.7
Duration of TB treatment						
<= 4 months	4/32	1.0		19/77	1.0	
> 4 months	9/68	1.1	0.4-3.2	55/361	0.6	0.4-0.9
Treatment is too long						
No	7/75	1.0		48/324	1.0	
Yes	6/25	2.9	0.9-9.9	26/114	1.7	1.0-2.9
Treatment discontinued once symptoms resolve						
No	9/88	1.0		64/391	1.0	
Yes	4/12	2.9	0.8-11	10/47	1.5	0.8-3.1
Know problems of stopping treatment						
Yes	7/68	1.0		49/316	1.0	
No	6/32	2.1	0.6-6.7	25/122	1.5	0.9-2.6
Confidence about completing treatment						
Somewhat/Very sure	2/8	1.0		2/22	1.0	
Not at all	11/92	0.5	0.1-1.8	72/416	1.8	0.5-7.0
Travel-related factors						
Travel to health center						
Walk	10/94	1.0		70/398	1.0	
Other	3/6	4.7	1.7-12	4/40	0.6	0.2-1.2
Travel a problem						
No	9/91	1.0		63/394	1.0	
Yes	4/9	7.1	1.6-31	11/44	1.8	0.8-3.9
Concern of transport						
No	4/12	1.0		9/51	1.0	
Some/ Very	9/88	4.3	1.1-17	65/387	1.1	0.5-2.4
Concern of Distance						
No	4/12	1.0		15/74	1.0	
Some/very	9/88	4.3	1.1-17	59/364	1.3	0.6-2.4
Concern of time						
No	5/16	1.0		14/67	1.0	
Some /very	8/84	4.2	1.2-15	60/371	1.3	0.7-2.6
Communication						
DOTS doctors tell problem of stopping						
Yes	7/71	1.0		57/363	1.0	
No	6/29	2.2	0.8-5.9	17/75	1.3	0.8-2.2
Where do you get most TB information						
DOTS center	9/82	1.0		66/395	1.0	
Other sources	4/18	2.0	0.7-5.8	8/43	1.2	0.6-2.2
Drug Supply						
Missed treatment due to no medicines						
No	13/100	1.0		67/422	1.0	
Yes	0/0			7/16	5.6	1.8-15

* N/T, number of subjects who are non-adherent / total subjects in that category

For the newly-diagnosed group, besides smoking and travel-related concerns, eight other factors (number of household members, chewing tobacco, considering treatment to be too long, treatment discontinued once symptoms resolve, knowing problems of stopping treatment, confidence about completing treatment, DOTS doctors informed the problems of stopping treatment, sources of TB information) qualified for inclusion in multivariable analysis. In this group, mode of travel to health center, considering travel a problem and concerns of access to transportation were grouped into a single variable (transport-related factor) and concerns of long distance to the DOTS center and loss of time from work were grouped into another variable (cost-related factor) as these variables were closely associated with each other. Among the residual other group, besides smoking and alcohol consumption during treatment and missing treatment due to lack of drug availability, two other factors (hiding disease from family and confidence about completing treatment) qualified for inclusion in the multivariable model.

Table 5 displays results of multivariable model where all qualifying risk factors were included for newly-diagnosed and the other residual group as appropriate. Among the newly-diagnosed patients smoking and only cost-related travel factor (concerns of long distance to DOTS center and loss of time from work) were positively associated with non-adherence to the treatment. Among the residual group, alcohol consumption during treatment and non-availability of drugs at the health center were significantly associated with non-adherence to the treatment.

**Table 5:** POR and 95% confidence intervals for non-adherence to treatment for potential risk factors, among newlydiagnosed and residual other groups, multivariable logistic regression models

Potential Risk factor	Newly-diagnosed		Others	
	
	aPOR*	95% C.I.	aPOR**	95% C.I.
Smoking	**7.8**	**1.2-49**	1.9	0.8-4.5
Transport-related travel factors	0.9	0.7-1.9	-	-
Cost-related travel factors	**5.1**	**1.4-19**	-	-
Alcohol	-	-	**3.6**	**1.5-8.3**
Missed Treatment due to no medicines	-	-	**5.1**	**1.6-16**
Household members	0.3	0.03-2.9	-	-
Tobacco chewing	1.6	0.3-8.2	-	-
Treatment is too long	1.4	0.3-7.4	-	-
Treatment can be discontinued once symptoms resolve	0.9	0.1-5.8	-	-
Know problems of stopping treatment	0.8	0.2-3.9	-	-
Not confidence about completing treatment	0.6	0.0-11	-	-
Health care provider did not explain problem of stopping treatment	2.8	0.5-14	-	-
Source of TB information	0.8	0.1-6.9	-	-
Hide disease from family	-	-	1.9	0.8-4.2
Confident about completing treatment	-	-	2.5	0.5-11

* aPOR for a risk factor adjusted for all the other variables in the model

## DISCUSSION

We found that 16% of patients among the total sample were non-adherent to the anti-TB therapy. Men were more likely to be non-adherent than women, but the difference was small. Among the subgroup of 100 newly-diagnosed patients, travel-related problems (concerns of distance and time) and smoking were statistically significantly associated with non-adherence. Among the residual other group, alcohol consumption and shortage of anti-TB drugs were significantly related to non-adherence. The overall non-adherence rate of 16% is within the range of non-adherence reported in other studies. Previous investigations conducted worldwide among patients receiving DOTS treatment have reported non-adherence rates ranging from 5% in Malawi^18^ to 29.8% in Zambia.^19^

### Newly-diagnosed group

#### Travel-related factors

Travel-related factors were major determinants of non-adherence among the newly-diagnosed patients. These patients are required to come to the DOTS centers three times a week during morning hours to receive medications. Of the several travel-related problems, long distances to the DOTS center and loss of time during travel were mainly related to non-adherence. These factors are indirectly related to cost. Patients may not have money to cover transportation cost and, being away from work would entail loss of wages. Among newly-diagnosed patients it is a challenge to balance the routine and the upheavals to the commitment to be adherent. Thus, it appears that provision of free treatment alone is not sufficient to facilitate adherence.

These findings are consistent with the results reported from studies conducted in other developing countries. In Madagascar, traveling problems to the health facility and concern of transportation cost were significantly associated with non-adherence to treatment.^9^ Two studies in Malaysia have also reported significant association between transportation problems and non-adherence.^7^,^8^

#### Smoking

We found smoking to be positively associated with non-adherence, among newly-diagnosed patients. A similar finding was observed among TB patients receiving standard TB regimen in Saudi Arabia^16^ and among TB patients receiving DOTS therapy in New York City, United States of America.^20^ None of the previous studies have suggested a possible reason why smoking was positively associated with non-adherence to the treatment.

### Residual other group

#### Alcoholism

We found alcohol consumption during the treatment period to be a risk factor for non-adherence especially in the residual group of patients. Alcohol consumption is extremely predominant among lower socio-economic class individuals in Mumbai and combined with deprivation of adequate nutrition is likely to lead to severe reactions like vomiting and nausea, thus promoting non-adherence to TB treatment among patients.

Our results are consistent with previous investigations.^14^,^15^ Alcoholism has been reported as a significant factor of patient non-adherence among tuberculosis patients receiving DOTS treatment in Denver, Colorado, USA.^14^

#### Shortage of adequate drug supply

We found that non-availability of drugs was positively associated with non-adherence among patients. In our study, 8% of non-adherent patients missed treatment due to non-availability of drugs. Although, this result is based on small numbers, it is important because under RNTCP as soon as a patient is registered with a clinic, a new box containing drug supply for the whole regimen is allocated for that patient. It is possible that drugs allocated to a patient are sometimes given to other patients due to lack of adequate drug-supply to cover all patients. This problem is related to administration of the program and would entail monitoring of staff on a periodic basis to ensure proper management practices. No other study has yet reported this finding.

#### Other factors

Previous investigations in rural Pakistan have found that inbred fears and supernatural beliefs were two major factors affecting adherence.^21^ However, we did not find fear and religious beliefs to affect adherence to treatment in our study. In our study, gender of the patient did not significantly affect non-adherence to treatment though previous reports have found that male sex of the patient significantly increased non-adherence to the treatment.^9^

The study has several strengths. There was a high participation rate (99.6%). Subjects were chosen by random sampling method, thus minimizing selection bias. We used objective procedures (patient treatment cards) to define outcome. Analytic procedures allowed control of various confounding factors.

### Limitations

The cross-sectional study design imposed several limitations. Due to short span of data collection and small samples in some of the subgroups it was difficult to obtain the equal representative sample in all subgroups. We were unable to contact a large proportion of lost to follow-up subjects (77%). This group may be distinctly different from other groups. Finally, findings may not be generalizable to the whole population of Mumbai, which is very diverse in socioeconomic status. However, it will be generalizable to the population attending the DOTS centers that generally belong to the lower socio-economic strata.

## CONCLUSION

In conclusion, this study evaluated the extent of adherence in pulmonary TB patients receiving DOTS therapy and various potential risk factors contributing to non-adherence. About one-fifth of the patients were non-adherent. Factors affecting non-adherence are different among newly-diagnosed patients and the residual other groups. Travel-related factors and smoking during treatment were significantly associated with non-adherence among newly-diagnosed patients, whereas alcohol consumption during treatment and missing drugs due to lack of adequate drug supply were factors responsible for non-adherence among the residual other groups. Currently, the RNTCP program of India has many components. Outreach workers, referral systems, regular patient follow-up and DOTS procedure are prominent features of the initiative. However, a more comprehensive approach, incorporating easier access to drugs, an ensured drug supply for every patient, effective solutions addressing travel-related concerns, modification of lifestyle behaviors and emphasizing on motivating patients to come to the clinic to receive therapy are essential to treatment completion among TB patients in an urban setting like Mumbai, India.
